# Interaction of Bacterial Exotoxins with Neutrophil Extracellular Traps: Impact for the Infected Host

**DOI:** 10.3389/fmicb.2016.00402

**Published:** 2016-03-30

**Authors:** Maren von Köckritz-Blickwede, Stefanie Blodkamp, Victor Nizet

**Affiliations:** ^1^Department of Physiological Chemistry, University of Veterinary Medicine HannoverHannover, Germany; ^2^Research Center for Emerging Infections and Zoonoses, University of Veterinary Medicine HannoverHannover, Germany; ^3^Department of Pediatric Pharmacology and Drug Discovery, San Diego School of Medicine, University of CaliforniaLa Jolla, CA, USA

**Keywords:** phagocyte extracellular traps, neutrophils, macrophages, leukocidin, cell death

## Abstract

Since their discovery in 2004, neutrophil extracellular traps (NETs) have been characterized as a fundamental host innate immune defense against various pathogens. Released in response to infectious and pro-inflammatory stimuli, NETs can immobilize invading pathogens within a fibrous matrix consisting of DNA, histones, and antimicrobial peptides. Conversely, excessive or dysregulated NET release may hold a variety of detrimental consequences for the host. A fine balance between NET formation and elimination is necessary to sustain a protective effect during infectious challenge. In recent years, a number of microbial virulence factors have been shown to modulate formation of NETs, thereby facilitating colonization or spread within the host. In this mini-review we summarize the contemporary research on the interaction of bacterial exotoxins with neutrophils that modulate NET production, focusing particular attention on consequences for the host. Understanding host–pathogen dynamics in this extracellular battlefield of innate immunity may provide novel therapeutic approaches for infectious and inflammatory disorders.

## Neutrophil Extracellular Traps

The formation of neutrophil extracellular DNA traps (so called NETs) was first recognized as a host innate immune defense mechanism against infections by Brinkmann et al. (2004). This discovery altered the fundamental conception of the innate immune function of phagocytes against pathogenic microbes in a most fascinating way. Whereas it was previously believed that neutrophils kill invading pathogens by intracellular uptake (phagocytosis) and subsequent killing, the discovery of NETs revealed an additional phagocytosis-independent mechanism. The released nuclear material including histones and DNA within the extracellular trap (ET) immobilize and occasionally kill several medically important bacteria, viruses, or parasites (Brinkmann et al., 2004).

Regarding the cellular pathways that lead to NET formation, most studies show that the cell in question undergoes NETosis, a process of death that differs morphologically from necrosis and apoptosis (Fuchs et al., 2007). In contrast to apoptotic or necrotic cells, cells that undergo NETosis show a prelytic decondensation of the chromatin associated with the disruption of the nuclear membrane. This disintegration of the nuclear membrane allows mixing of DNA and histones with granule components, which are then extracellularly released as web-like fibers with a diameter of approximately 15 to 17 nm, associated with globular proteins or antimicrobial peptides. NET-based antimicrobial activity remains active when cells are treated with the actin microfilament inhibitor cytochalasin D, indicating that this phenomenon is independent of phagocytosis. However, the antimicrobial activity of NETs can be abolished when NETs are treated with DNase, confirming that DNA comprises the functional backbone of the released fibers (Brinkmann et al., 2004; Fuchs et al., 2007).

The formation of NETs was initially thought to be an antimicrobial defense strategy and cell death pathway specific for neutrophils (Brinkmann et al., 2004). However, von Köckritz-Blickwede et al. (2008) mast cells were shown to also deploy mast cell extracellular traps (MCETs) in defense against bacterial pathogens. During recent years, further evidence has accumulated demonstrating ET formation occurs in eosinophils (Yousefi et al., 2008), basophils (Schorn et al., 2012), fibrocytes (Kisseleva et al., 2011), macrophages (Bartneck et al., 2010; Chow et al., 2010), and monocytes (Bartneck et al., 2010; Chow et al., 2010).

It is important to mention that some authors have demonstrated experimentally that eosinophils and neutrophils can release antimicrobial ETs in response to infection while remaining in a viable status (Yousefi et al., 2008, 2009). NET release by viable cells was confirmed *in vivo* in a murine model of *Staphylococcus aureus* skin infection (Yipp et al., 2012). Here, NET formation was seen as a dynamic process, which occurs by vesicular release of nuclear material during migration of neutrophils through the tissue. It is still unclear, however, how such budding and final release of nuclear material is initiated. Thus, based on current knowledge it seems that at least two different mechanisms can lead to NET formation: a viable form involving vesicular release and the more well-understood NETosis form associated with rupture of the nuclear membrane. Research has begun to explore how certain drugs, including statins (Chow et al., 2010) and tamoxifen (Corriden et al., 2015), modulate neutrophil functions to accentuate NET formation, which could be beneficial as an adjunctive therapy for extracellular pathogens such as *S. aureus* that efficiently avoid phagocytic killing by neutrophils.

In contrast to their protective effect against several infections, there is increasing evidence that a dysregulated NET release can provoke autoimmune reactions, tissue damage and impaired cellular functions (Villanueva et al., 2011; Saffarzadeh et al., 2012). Unchecked, aberrant NET formation can result in pathological damage as vascular thrombosis (Fuchs et al., 2010) or chronic lung inflammation in cystic fibrosis (Papayannopoulos et al., 2011). Countermeasures to the excessive NET formation may be of therapeutic utility in such cases as for example DNAse treatment in patients with cystic fibrosis (Fuchs et al., 1994) or the usage of anti-histone antibodies to alleviate vascular thrombosis (Semeraro et al., 2011). Since sustained blockade of NET formation may carry a risk of increased susceptibility to certain infections, anti-NET therapy might be targeted to severe autoimmune or inflammatory diseases, when pro-inflammatory activities of NETs outweigh their protective benefits (Saffarzadeh and Preissner, 2013).

## The Role of NETs Against Infection

In several studies, NETs were found to play a protective role against various infecting organisms, often employing mutagenesis of key evasion factors of the respective organisms. It is thought that NETs can act in two ways: (1) pathogen immobilization and (2) growth inhibition or killing of the microbe. The relative importance of killing is a point of some contention in the literature (Menegazzi et al., 2012). However, based on vital immunofluorescent staining of bacteria with DNA intercalating dyes, microbial killing by NETs has been demonstrated definitively by several authors (Lauth et al., 2009; Berends et al., 2010; Halverson et al., 2015). However, since the overall killing potential of NETs is physically restricted, the cumulative effect of NETs may be functionally bacteriostatic (Baums and von Köckritz-Blickwede, 2015).

**FIGURE 1:**
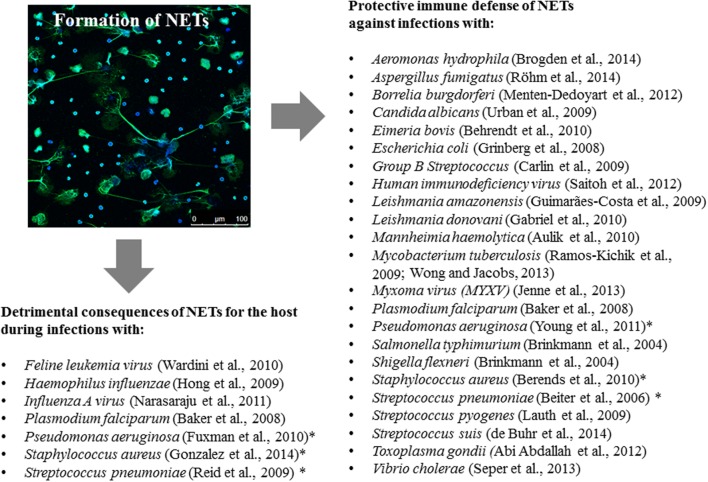
**Extracellular trap formation during infections: consequences for the host.** The figure summarizes the protective versus detrimental role of NETs for the host during an infection with respective pathogens. In some cases (highlighted with an asterisk), there is benefit and harm depending on site, timing and magnitude of infection. The immunofluorescence micrograph is showing the release of NETs from human blood-derived neutrophils. NETs were stained with antibodies against histone-DNA complexes (green) and DAPI to stain the nuclei and nuclear DNA (blue).

Because some bacteria have evolved highly efficient resistance strategies against antimicrobial activity of NETs, in some specific circumstances, NETs can help to establish an infectious niche. For example, in the case of a primary influenza A infection of the middle ear which boosts formation of NETs by infiltrating neutrophils, resistant *Streptococcus pneumoniae* can use those NETs to multiply and persist in the middle ear cavity (Short et al., 2014). Additionally, some studies show that *S. pneumoniae* and *Haemophilus influenzae* can incorporate NETs into biofilms that promote their persistence in the host (Hong et al., 2009; Reid et al., 2009). In the end, it depends on the pathogen, its array of immune resistance factors and the anatomical site of infection, as to whether NETs can serve a protective function for the host during an infection (**Figure 1**).

## Categories of Bacterial NET Evasion Factors

Neutrophil extracellular trap evasion factors can be classified in three different phenotypes: (1) NET degradation, (2) resistance to the intrinsic antimicrobial effectors within NETs, or (3) the suppression of NET production (von Köckritz-Blickwede and Nizet, 2009). The best-studied NET evasion factor is the activity of microbial nucleases that degrade NETs deployed by innate immune cells to escape entrapment. Membrane-bound or released nucleases have been shown to be expressed by the following microbes as NET evasion factors: *Aeromonas hydrophila* (Brogden et al., 2012), *Escherichia coli* (Möllerherm et al., 2015), *Leptospira* sp. (Scharrig et al., 2015), *Neisseria gonorhoeae* (Juneau et al., 2015), *S. aureus* (Berends et al., 2010), *S. agalactiae* (Derré-Bobillot et al., 2013), *S. pneumoniae* (Beiter et al., 2006), *S. pyogenes* (Buchanan et al., 2006; Chang et al., 2011), *Streptococcus synguinis* (Morita et al., 2014), *S. suis* (de Buhr et al., 2014), *Vibrio cholera* (Seper et al., 2013), and *Yersinia enterocolitica* (Möllerherm et al., 2015). Whereas, resistance to antimicrobial effectors in ETs is mainly mediated by surface-bound virulence factors, e.g., exopolysaccharide capsules in case of *S. pyogenes* (Cole et al., 2010) or *S. pneumonia* (Wartha et al., 2007), the suppression of NET formation is mainly carried out by released factors including proteases (Zinkernagel et al., 2008).

## Bacterial Exotoxins that Modulate NET Formation

During the last 10 years, there is increasing evidence in the literature that different exotoxins released by Gram-positive or Gram-negative bacteria are able to modulate the process of NET formation (**Table 1**): to date, at least 11 different exotoxins have been published to modulate NET formation, with nine of them reported to enhance NET formation while two suppress the process (**Table 1**). First we will set our attention on those toxins that induce NET formation.

**Table 1:** Effect of bacterial exotoxins on formation of extracellular traps.

Microbe	Exoprotein	Effect on cells	Mechanism	Cell death	Putative consequences for the host	Reference
*Bordetella pertussis*	Adenylate cyclase toxin (ACT)	Suppression of NET formation and apoptosis	Generation of cAMP and inhibition of oxidative burst	Impairment of cell lysis	ACT promotes neutrophil infiltration by inhibiting neutrophil death	Eby et al., 2014
*Escherichia coli*	Hemolysin	Induction of ETs in murine and human macrophages	Not known	Not known	Not known	Aulik et al., 2012
*Mannheimia haemolytica*	Leukotoxin	Induction of ETs in bovine neutrophils, as well as bovine, murine, and human macrophages	CD18- and NADPH-oxidase dependent	Delayed LDH release, no necrosis, no aptoptosis	Trapping and killing of *M. haemolytica*	Aulik et al., 2010, 2012
*Mycobacterium tuberculosis*	Early secretory antigen-6 protein	Induction of secondary necrosis and ET formation of phosphatidylserine externalized neutrophils	Intracellular Ca^2+^ overload	Cell lysis	Necrotic granulomas during tuberculosis	Francis et al., 2014
*Pseudomonas aeruginosa*	Pyocyanin	Induction of NETs	NADPH- oxidase- and Jun N-terminal kinase-dependent	Cell lysis	Inflammatory condition during cystic fibrosis	Rada et al., 2013
*Staphylococcus aureus*	N-terminal ArgD peptides	Induction of NETs	Unknown	Cell lysis	Aggravation of skin lesions	Gonzalez et al., 2014
*Staphylococcus aureus*	Leukotoxin GH	Induction of NETs	Non-specific cytolysis	Non-specific cytolysis	Entrapment of *S. aureus*	Malachowa et al., 2013
*Staphylococcus aureus*	Panton-Valentin-leukocidin	Induction of rapid nuclear NET formation	Vesicular release of nuclear DNA, independent of NADPH- oxidase	No cell lysis	Entrapment of *S. aureus*	Pilsczek et al., 2010
*Staphylococcus epidermidis*	Phenol-soluble-modulin γ (δ-toxin)	Induces NET formation and colocalizes with NETs and host antimicrobial peptides	Physically binding to host derived antimicrobial peptides and DNA	Cell lysis	Cooperates with host antimicrobial peptides against bacterial pathogens	Cogen et al., 2010
*Streptocococus pyogenes*	M1 protein	Induces formation of NETs and MCETs	Unknown	Cell lysis	Trapping and killing of *S. pyogenes*	Lauth et al., 2009; Oehmcke et al., 2009
*Streptocococus pyogenes*	Streptolysin O	Suppression of NET formation	Impairment of oxidative burst	Sublytic con-centrations	Severe infection	Uchiyama et al., 2015

The M1-protein of the Gram-positive bacterium *S. pyogenes*, also known as group A *Streptococcus* (GAS), was the first exotoxin described to induce formation of ETs by neutrophils and mast cells (Lauth et al., 2009). The surface-anchored M1 protein is a classical virulence factor that promotes resistance to phagocytosis. However, after proteolytic release from the *S. pyogenes* surface, the M1 protein forms a proinflammatory supramolecular network with fibrinogen that contributes to the pathophysiology of streptococcal toxic shock-like syndrome (Herwald et al., 2004). Two parallel studies showed in 2009, that the M1 protein is able to mediate formation of NETs as well as MCETs, and thereby contribute to entrapment and killing of the bacteria (Lauth et al., 2009; Oehmcke et al., 2009). Besides induction of ETs, M1 protein promotes resistance to the human cathelicidin antimicrobial peptide LL-37, an important effector of bacterial killing by neutrophils and mast cells (Lauth et al., 2009; LaRock et al., 2015).

*Staphylococcus epidermidis* is a common colonizer of healthy human skin and is hypothesized to play a mutually beneficial role in the cutaneous niche. Cogen et al. (2010) demonstrated that *S. epidermidis* phenol-soluble-modulin γ (δ-toxin) boosts NET production and further colocalizes with endogenous host antimicrobial peptides within the architecture of the expressed NETs. In antimicrobial assays against the pathogen *S. pyogenes*, δ-toxin cooperated with host-derived antimicrobial peptides cathelicidins (human LL-37 and murine CRAMP) and human beta-defensins (hBD2 and hBD3) to kill the pathogenic streptococci. Coimmunoprecipitation and tryptophan spectroscopy demonstrated direct binding of δ-toxin to LL-37, CRAMP, hBD2, and hBD3, as well as DNA. These data suggest that commensal *S. epidermidis*-derived δ-toxin cooperates with the host-derived antimicrobial peptides in the innate immune system to reduce survival of an important human bacterial pathogen. This could be corroborated in a mouse wound model where *S. pyogenes* survival was reduced when the wounds were pretreated with δ-toxin. These data described a novel therapeutic application of a bacterial toxin against pathogenic bacteria to enhance formation of NETs and support the host innate immune system.

*Mannheimia haemolytica* leukotoxin (LKT) induces formation of extracellular traps in bovine neutrophils and macrophages (Aulik et al., 2010, 2012). LKT is a member of the repeats-in-toxin (RTX) family of exoproteins produced by a wider variety of Gram-negative bacteria. In addition to the native fully active LKT, its non-cytolytic pro-LKT precursor also stimulated macrophage extracellular traps (METs) formation. Formation of METs in response to LKT required NADPH oxidase activity, as previously demonstrated for NETosis. METs induced in response to LKT trapped and killed a portion of the toxin-producing *M. haemolytica* cells. In contrast to NETosis, which is reported to occur between 10 min to 4 h after pathogen exposure, LKT-induced MET formation was extremely rapid, with significant accumulation of extracellular DNA within 2 min after macrophage stimulation.

Interestingly, the LKT-mediated cytotoxic effects are specific to ruminants, since LKT binds to amino acids 5–17 of the signal sequence of CD18, which is not present on mature leukocytes from humans and other mammalian species (Shanthalingam and Srikumaran, 2009). CD18 was confirmed by Aulik et al. (2010) to play a role in LKT-dependent NET formation in bovine phagocytes. Similarly, to the LKT, the related RTX family toxin, uropathgenic *E. coli* (UPEC) hemolysin, induced NET formation in the mouse and human monocyte/macrophage cell lines indicating that NET induction may be a general phenomenon in response to active RTX toxins (Aulik et al., 2012). At this point it remains difficult to assess whether NET production in response to RTX toxins leads to a protective host defense against *M. haemolytica* or other Gram-negative bacteria. In the case of LKT, bacterial cells entrapped in NETs can continue to secrete LKT, an important virulence determinant, which could exacerbate lung inflammation (Aulik et al., 2010, 2012).

The *Pseudomonas aeruginosa* virulence factor pyocyanin stimulates the release of NETs in a NADPH oxidase-dependent manner (Rada et al., 2013). Pyocyanin is a redox-active pigment associated with diminished lung function in cystic fibrosis. In cystic fibrosis airways, *P. aeruginosa* resides in biofilms protected from neutrophil phagocytic activity or from entrapment by NETs (Rada et al., 2013). The authors speculate that enhanced ROS-dependent NET formation by *P. aeruginosa* pyocyanin contributes to inflammatory conditions observed in chronically infected cystic fibrosis airways. Parenthetically, chronic granulomatous disease (CGD) patients, whose neutrophils are unable to make NETs, are not recognized to be disproportionately susceptible to infections with *Pseudomonas* species (Rada and Leto, 2008).

A specific activity of an exotoxin was shown for the *Mycobacterium tuberculosis* leukocidin ESAT-6, which induced NET formation only in a subpopulation of neutrophils with externalized phosphatidylserine as a marker for apoptosis (Francis et al., 2014). Thus, ESAT-6 induced NET formation was similar to secondary necrosis of pre-activated neutrophils and dependent on Ca^2+^. In this case, increased NET formation was also speculated to contribute to virulence of tuberculosis, since it was recently observed that the development of necrotic granulomas in a mouse model of progressive tuberculosis were associated with the presence of extracellular bacteria, neutrophil necrosis and NET-like structures (Francis et al., 2014).

In the case of the leading human pathogen *S. aureus*, three different exotoxins enhance the release of NETs: N-terminal AgrD peptides (Gonzalez et al., 2014), leukotoxin GH (Malachowa et al., 2013), also known as LukAB (Dumont et al., 2011), thus named here LukGH/AB, and Panton-Valentin leukocidin (PVL; Pilsczek et al., 2010). AgrD is the precursor for the auto-inducing peptide in a well-known quorum sensing system regulating virulence phenotypes of *S. aureus*. A recent mass spectrometry-based study identified formylated and non-formylated peptide variants derived from AgrD N-terminal leader domain in *S. aureus* cell-free culture supernatant to act cytotoxic, modulate neutrophil chemotaxis and induce formation of NETs. As a consequence a detrimental effect for the host was hypothesized as indicated by aggravation of skin lesions *in vivo* in a murine model. The cellular pathways mediating this process of AgrD induced NET formation remain unclear (Gonzalez et al., 2014).

*Staphylococcus aureus* LukGH/AB, also promotes formation of NETs in association with death of the neutrophil (Malachowa et al., 2013). LukGH/AB is a pore-forming cytolytic toxin with proinflammatory properties, similar to those established for PVL. But unlike PVL, LukGH/AB did not prime human neutrophils for increased production of reactive oxygen species nor did it enhance binding and/or uptake of *S. aureus*. LukGH/AB promoted the release of NETs, which in turn ensnared but did not achieve killing of *S. aureus*. These authors found that electropermeabilization of human neutrophils, used to create pores in the neutrophil plasma membrane, induced NET formation in a similar way. This finding indicated that NETs can be generated during non-specific cytolysis.

The above phenomemon stands in contrast to NET formation initiated by PVL production in *S. aureus*. Pilsczek et al. (2010) demonstrated a novel rapid (5–60 min) process of NET formation that did not require cell lysis or even breach of the plasma membrane. The authors show that neutrophils treated with *S. aureus* supernatant show rounded and condensed nuclei, followed by the separation of the inner and outer nuclear membrane and budding of vesicles filled with nuclear DNA. The vesicles are extruded intact into extracellular space where they rupture, release the chromatin and form NETs. PVL was identified as one key effector of NET formation present in the *S. aureus* supernatant, but other yet unidentified molecules aside from PVL cannot be excluded as contributors to NET production. This rapid process of NET formation against *S. aureus* was dubbed “dynamic NETosis” and was recently proven to serve as an efficient host defense strategy during *S. aureus* skin infections in man and mice (Yipp et al., 2012). Since, PVL targets a different receptor on the neutrophil compared to the LukGH/AB, namely the C5aR receptor (Spaan et al., 2013) and the CD11b receptor (Dumont et al., 2013), respectively, it still remains to be determined if the receptor-mediated signaling leads to the different NET phenotypes.

Two toxins have been described so far to suppress NET formation, perhaps representing an immune evasion strategy by the respective pathogens: *Bordetella pertussis* adenylate cyclase toxin (Eby et al., 2014) and *S. pyogenes* streptolysin O (SLO; Uchiyama et al., 2015). Both mechanisms are coupled to suppression of neutrophil oxidative burst. In case of *S. pyogenes* SLO, this mechanism plays a key role of SLO in the pathogen’s resistance to immediate neutrophil killing. In case of *B. pertussis*, the relevance of NET inhibition by the toxin is still unclear, since it is not known whether or not NETs are able to entrap and clear *B. pertussis*. This study highlighted how convalescent phase serum from humans following clinical pertussis blocked the ACT-mediated suppression of NET formation (Eby et al., 2014). Based on this finding, the authors mention that their data should alert investigators to the ability for ACT, and antibodies to ACT, to dysregulate neutrophil death mechanisms and potentially influence local tissue damage during infection with *B. pertussis*.

## Concluding Comments

With increasing publications in the NET field it is well-known that NETs are on one hand protective against several infections, but may also result in detrimental effects when released in excessive amount. The complexity may be compared with the release of cytokines during infection and inflammation, which serve an essential role in an efficient immune response of the host against infections, but in which overwhelming cytokine storm can lead to septic shock and accelerate death of the host. The cumulative balance between NET formation and NET degradation – similar to cytokine release – defines the protective versus detrimental effects on the organism. Currently, it is under discussion in the literature if and how NETs can act as a novel therapeutic or prophylactic target for boosting immunity to bacterial infections or mitigating inflammatory diseases associated with detrimental NET formation. To rationally and effectively pharmacologically interfere with the process of NET formation, the cellular processes mediating this phenomenon need to be understood more in detail. Identification of bacterial factors, especially exotoxins, with specific roles in NET modulation, may serve as probes to understand the molecular basis of NET generation and antimicrobial activity. Furthermore, exotoxins might themselves harbor therapeutic potential when their effects against the host cells are fully characterized.

## Author Contributions

SB collected the literature, MK-B and VN wrote the manuscript. All authors have proofread the manuscript.

## Conflict of Interest Statement

The authors declare that the research was conducted in the absence of any commercial or financial relationships that could be construed as a potential conflict of interest.

## References

[B1] Abi Abdallah D. S. Lin C. Ball C. J. King M. R. Duhamel G. E. Denkers E. Y. (2012). Toxoplasma gondii triggers release of human and mouse neutrophil extracellular traps. *Infect. Immun.* 80 768–777. 10.1128/IAI.05730-5711 22104111 PMC3264325

[B2] Aulik N. A. Hellenbrand K. M. Czuprynski C. J. (2012). *Mannheimia haemolytica* and its leukotoxin cause macrophage extracellular trap formation by bovine macrophages. *Infect. Immun.* 80 1923–1933. 10.1128/IAI.06120-11 22354029 PMC3347434

[B3] Aulik N. A. Hellenbrand K. M. Klos H. Czuprynski C. J. (2010). *Mannheimia haemolytica* and its leukotoxin cause neutrophil extracellular trap formation by bovine neutrophils. *Infect. Immun.* 78 4454–4466. 10.1128/IAI.00840-10 20823211 PMC2976348

[B4] Baker V. S. Imade G. E. Molta N. B. Tawde P. Pam S. D. Obadofin M. O. (2008). Cytokine-associated neutrophil extracellular traps and antinuclear antibodies in *Plasmodium falciparum* infected children under six years of age. *Malar. J.* 7:41 10.1186/1475-2875-7-41 PMC2275287 18312656

[B5] Bartneck M. Keul H. A. Zwadlo-Klarwasser G. Groll J. (2010). Phagocytosis independent extracellular nanoparticle clearance by human immune cells. *Nano Lett.* 10 59–63. 10.1021/nl902830x 19994869

[B6] Baums C. G. von Köckritz-Blickwede M. (2015). Novel role of DNA in neutrophil extracellular traps. *Trends Microbiol.* 23 330–331. 10.1016/j.tim.2015.04.003 25913613

[B7] Behrendt J. H. Ruiz A. Zahner H. Taubert A. Hermosilla C. (2010). Neutrophil extracellular trap formation as innate immune reactions against the apicomplexan parasite Eimeria bovis. *Vet. Immunol. Immunopathol.* 133 1–8. 10.1016/j.vetimm.2009.06.012 19625090

[B8] Beiter K. Wartha F. Albiger B. Normark S. Zychlinsky A. Henriques-Normark B. (2006). An endonuclease allows *Streptococcus pneumoniae* to escape from neutrophil extracellular traps. *Curr. Biol.* 16 401–407. 10.1016/j.cub.2006.01.056 16488875

[B9] Berends E. T. Horswill A. R. Haste N. M. Monestier M. Nizet V. von Köckritz-Blickwede M. (2010). Nuclease expression by *Staphylococcus aureus* facilitates escape from neutrophil extracellular traps. *J. Innate Immun.* 2 576–586. 10.1159/000319909 20829609 PMC2982853

[B10] Brinkmann V. Reichard U. Goosmann C. Fauler B. Uhlemann Y. Weiss D. S. (2004). Neutrophil extracellular traps kill bacteria. *Science* 303 1532–1535. 10.1126/science.1092385 15001782

[B11] Brogden G. Krimmling T. Adamek M. Naim H. Y. Steinhagen D. von Köckritz-Blickwede M. (2014). The effect of β-glucan on formation and functionality of neutrophil extracellular traps in carp (*Cyprinus carpio* L.). *Dev. Comp. Immunol.* 44 280–285. 10.1016/j.dci.2014.01.003 24434196

[B12] Brogden G. von Köckritz-Blickwede M. Adamek M. Reuner F. Jung-Schroers V. Naim H. Y. (2012). β-Glucan protects neutrophil extracellular traps against degradation by *Aeromonas hydrophila* in carp (*Cyprinus carpio*). *Fish Shellfish Immunol.* 33 1060–1064. 10.1016/j.fsi.2012.08.009 22959188

[B13] Buchanan J. T. Simpson A. J. Aziz R. K. Liu G. Y. Kristian S. A. Kotb M. (2006). DNase expression allows the pathogen group a Streptococcus to escape killing in neutrophil extracellular traps. *Curr. Biol.* 16 396–400. 10.1016/j.cub.2005.12.039 16488874

[B14] Carlin A. F. Uchiyama S. Chang Y. C. Lewis A. L. Nizet V. Varki A. (2009). Molecular mimicry of host sialylated glycans allows a bacterial pathogen to engage neutrophil Siglec-9 and dampen the innate immune response. *Blood* 113 3333–3336. 10.1182/blood-2008-11-187302 19196661 PMC2665898

[B15] Chang A. Khemlani A. Kang H. Proft T. (2011). Functional analysis of *Streptococcus pyogenes* nuclease A (SpnA), a novel group A streptococcal virulence factor. *Mol. Microbiol.* 79 1629–1642. 10.1111/j.1365-2958.2011.07550.x 21231972

[B16] Chow O. A. Köckritz-Blickwede M. von Bright A. T. Hensler M. E. Zinkernagel A. S. Cogen A. L. (2010). Statins enhance formation of phagocyte extracellular traps. *Cell Host Microbe* 8 445–454. 10.1016/j.chom.2010.10.005 21075355 PMC3008410

[B17] Cogen A. L. Yamasaki K. Muto J. Sanchez K. M. Crotty Alexander L. Tanios J. (2010). Staphylococcus epidermidis antimicrobial delta-toxin (phenol-soluble modulin-gamma) cooperates with host antimicrobial peptides to kill group a Streptococcus. *PLoS ONE* 5:e8557 10.1371/journal.pone.0008557 PMC2796718 20052280

[B18] Cole J. N. Pence M. A. von Köckritz-Blickwede M. Hollands A. Gallo R. L. Walker M. J. (2010). M protein and hyaluronic acid capsule are essential for in vivo selection of covRS mutations characteristic of invasive serotype M1T1 group A Streptococcus. *MBio* 1:e191 10.1128/mBio.00191-10 PMC2934611 20827373

[B19] Corriden R. Hollands A. Olson J. Derieux J. Lopez J. Chang J. T. (2015). Tamoxifen augments the innate immune function of neutrophils through modulation of intracellular ceramide. *Nat. Commun.* 13:8369 10.1038/ncomms9369 PMC4610010 26458291

[B20] de Buhr N. Neumann A. Jerjomiceva N. Köckritz-Blickwede M. Baums C. G. (2014). *Streptococcus suis* DNase SsnA contributes to degradation of neutrophil extracellular traps (NETs) and evasion of NET-mediated antimicrobial activity. *Microbiology* 160 385–395. 10.1099/mic.0.072199-0 24222615

[B21] Derré-Bobillot A. Cortes-Perez N. G. Yamamoto Y. Kharrat P. Couvé E. Da Cunha V. (2013). Nuclease A (Gbs0661), an extracellular nuclease of *Streptococcus agalactiae*, attacks the neutrophil extracellular traps and is needed for full virulence. *Mol. Microbiol.* 89 518–531. 10.1111/mmi.12295 23772975

[B22] Dumont A. L. Nygaard T. K. Watkins R. L. Smith A. Kozhaya L. Kreiswirth B. (2011). Characterization of a new cytotoxin that contributes to *Staphylococcus aureus* pathogenesis. *Mol. Microbiol.* 79 814–825. 10.1111/j.1365-2958.2010.07490.x 21255120 PMC3312031

[B23] Dumont A. L. Yoong P. Day C. J. Alonzo F. III McDonald W. H. Jennings M. P. (2013). *Staphylococcus aureus* LukAB cytotoxin kills human neutrophils by targeting the CD11b subunit of the integrin Mac-1. *Proc. Natl. Acad. Sci. U.S.A.* 110 10794–10799. 10.1073/pnas.1305121110 23754403 PMC3696772

[B24] Eby J. C. Gray M. C. Hewlett E. L. (2014). Cyclic AMP-mediated suppression of neutrophil extracellular trap formation and apoptosis by the *Bordetella pertussis* adenylate cyclase toxin. *Infect. Immun.* 82 5256–5269. 10.1128/IAI.02487-14 25287922 PMC4249293

[B25] Francis R. J. Butler R. E. Stewart G. R. (2014). *Mycobacterium tuberculosis* ESAT-6 is a leukocidin causing Ca^2+^ influx, necrosis and neutrophil extracellular trap formation. *Cell Death Dis.* 5:e1474 10.1038/cddis.2014.394 PMC4237235 25321481

[B26] Fuchs H. J. Borowitz D. S. Christiansen D. H. Morris E. M. Nash M. L. Ramsey B. W. (1994). Effect of aerosolized recombinant human DNase on exacerbations of respiratory symptoms and on pulmonary function in patients with cystic fibrosis. The pulmozyme study group. *N. Engl. J. Med.* 331 637–642. 10.1056/NEJM199409083311003 7503821

[B27] Fuchs T. A. Abed U. Goosmann C. Hurwitz R. Schulze I. Wahn V. (2007). Novel cell death program leads to neutrophil extracellular traps. *J. Cell Biol.* 176 231–241. 10.1083/jcb.200606027 17210947 PMC2063942

[B28] Fuchs T. A. Brill A. Duerschmied D. Schatzberg D. Monestier M. Myers D. D. Jr. (2010). Extracellular DNA traps promote thrombosis. *Proc. Natl. Acad. Sci. U.S.A.* 107 15880–15885. 10.1073/pnas.1005743107 20798043 PMC2936604

[B29] Fuxman Bass J. I. Russo D. M. Gabelloni M. L. Geffner J. R. Giordano M. Catalano M. (2010). Extracellular DNA: a major proinflammatory component of *Pseudomonas aeruginosa* biofilms. *J. Immunol.* 184 6386–6395. 10.4049/jimmunol.0901640 20421641

[B30] Gabriel C. McMaster W. R. Girard D. Descoteaux A. (2010). *Leishmania donovani* promastigotes evade the antimicrobial activity of neutrophil extracellular traps. *J. Immunol.* 185 4319–4327. 10.4049/jimmunol.1000893 20826753

[B31] Gonzalez D. J. Corriden R. Akong-Moore K. Olson J. Dorrestein P. C. Nizet V. (2014). N-terminal ArgD peptides from the classical *Staphylococcus aureus* Agr system have cytotoxic and proinflammatory activities. *Chem. Biol.* 21 1457–1462. 10.1016/j.chembiol.2014.09.015 25457179 PMC4304878

[B32] Grinberg N. Elazar S. Rosenshine I. Shpigel N. Y. (2008). Beta-hydroxybutyrate abrogates formation of bovine neutrophil extracellular traps and bactericidal activity against mammary pathogenic *Escherichia coli*. *Infect. Immun.* 76 2802–2807. 10.1128/IAI.00051-08 18411287 PMC2423099

[B33] Guimarães-Costa A. B. Nascimento M. T. Froment G. S. Soares R. P. Morgado F. N. Conceição-Silva F. (2009). *Leishmania amazonensis* promastigotes induce and are killed by neutrophil extracellular traps. *Proc. Natl. Acad. Sci. U.S.A.* 106 6748–6753. 10.1073/pnas.0900226106 19346483 PMC2672475

[B34] Halverson T. W. Wilton M. Poon K. K. Petri B. Lewenza S. (2015). DNA is an antimicrobial component of neutrophil extracellular traps. *PLoS Pathog.* 11:e1004593 10.1371/journal.ppat.1004593 PMC4295883 25590621

[B35] Herwald H. Cramer H. Mörgelin M. Russell W. Sollenberg U. Norrby-Teglund A. (2004). M protein, a classical bacterial virulence determinant, forms complexes with fibrinogen that induce vascular leakage. *Cell* 116 367–379. 10.1016/S0092-8674(04)00057-1 15016372

[B36] Hong W. Juneau R. A. Pang B. Swords W. E. (2009). Survival of bacterial biofilms within neutrophil extracellular traps promotes nontypeable *Haemophilus influenzae* persistence in the chinchilla model for otitis media. *J. Innate Immun.* 1 215–224. 10.1159/000205937 20375579 PMC6951045

[B37] Jenne C. N. Wong C. H. Zemp F. J. McDonald B. Rahman M. M. Forsyth P. A. (2013). Neutrophils recruited to sites of infection protect from virus challenge by releasing neutrophil extracellular traps. *Cell Host Microbe.* 13 169–180. 10.1016/j.chom.2013.01.005 23414757

[B38] Juneau R. A. Stevens J. S. Apicella M. A. Criss A. K. (2015). A thermonuclease of *Neisseria gonorrhoeae* enhances bacterial escape from killing by neutrophil extracellular traps. *J. Infect. Dis.* 212 316–324. 10.1093/infdis/jiv031 25605868 PMC4490236

[B39] Kisseleva T. Köckritz-Blickwede M. Reichart D. McGillvray S. M. Wingender G. Kronenberg M. (2011). Fibrocyte-like cells recruited to the spleen support innate and adaptive immune responses to acute injury or infection. *J. Mol. Med.* 89 997–1013. 10.1007/s00109-011-0756-0 21499735 PMC3171633

[B40] LaRock C. N. Döhrmann S. Todd J. Corriden R. Olson J. Johannssen T. (2015). Group a streptococcal M1 protein sequesters cathelicidin to evade innate immune killing. *Cell Host Microbe* 18 471–477. 10.1016/j.chom.2015.09.004 26468750 PMC4636435

[B41] Lauth X. von Köckritz-Blickwede M. McNamara C. W. Myskowski S. Zinkernagel A. S. Beall B. (2009). M1 protein allows Group A streptococcal survival in phagocyte extracellular traps through cathelicidin inhibition. *J. Innate Immun.* 1 202–214. 10.1159/000203645 20375578 PMC3241932

[B42] Malachowa N. Kobayashi S. D. Freedman B. Dorward D. W. DeLeo F. R. (2013). *Staphylococcus aureus* leukotoxin GH promotes formation of neutrophil extracellular traps. *J. Immunol.* 191 6022–6029. 10.4049/jimmunol.1301821 24190656 PMC3903389

[B43] Menegazzi R. Decleva E. Dri P. (2012). Killing by neutrophil extracellular traps: fact or folklore? *Blood* 119 1214–1216. 10.1182/blood-2011-07-364604 22210873

[B44] Menten-Dedoyart C. Faccinetto C. Golovchenko M. Dupiereux I. Van Lerberghe P. B. Dubois S. (2012). Neutrophil extracellular traps entrap and kill *Borrelia burgdorferi* sensu stricto spirochetes and are not affected by *Ixodes ricinus* tick saliva. *J. Immunol.* 189 5393–5501. 10.4049/jimmunol.1103771 23109724

[B45] Möllerherm H. Neumann A. Schilcher K. Blodkamp S. Zeitouni N. E. Dersch P. (2015). *Yersinia enterocolitica*-mediated degradation of neutrophil extracellular traps (NETs). *FEMS Microbiol. Lett.* 362:fnv192 10.1093/femsle/fnv192 26459885

[B46] Morita C. Sumioka R. Nakata M. Okahashi N. Wada S. Yamashiro T. (2014). Cell wall-anchored nuclease of *Streptococcus sanguinis* contributes to escape from neutrophil extracellular trap-mediated bacteriocidal activity. *PLoS ONE* 9:e103125 10.1371/journal.pone.0103125 PMC4118848 25084357

[B47] Narasaraju T. Yang E. Samy R. P. Ng H. H. Poh W. P. Liew A. A. (2011). Excessive neutrophils and neutrophil extracellular traps contribute to acute lung injury of influenza pneumonitis. *Am. J. Pathol.* 179 199–210. 10.1016/j.ajpath.2011.03.013 21703402 PMC3123873

[B48] Oehmcke S. Mörgelin M. Herwald H. (2009). Activation of the human contact system on neutrophil extracellular traps. *J. Innate. Immun.* 1 225–230. 10.1159/000203700 20375580 PMC6951039

[B49] Papayannopoulos V. Staab D. Zychlinsky A. (2011). Neutrophil elastase enhances sputum solubilization in cystic fibrosis patients receiving DNase therapy. *PLoS ONE.* 6:e28526 10.1371/journal.pone.0028526 PMC3235130 22174830

[B50] Pilsczek F. H. Salina D. Poon K. K. Fahey C. Yipp B. G. Sibley C. D. (2010). A novel mechanism of rapid nuclear neutrophil extracellular trap formation in response to Staphylococcus aureus. *J. Immunol.* 185 7413–7425. 10.4049/jimmunol.1000675 21098229

[B51] Rada B. Jendrysik M. A. Pang L. Hayes C. P. Yoo D. G. Park J. J. (2013). Pyocyanin-enhanced neutrophil extracellular trap formation requires the NADPH oxidase. *PLoS ONE* 8:e54205 10.1371/journal.pone.0054205 PMC3544820 23342104

[B52] Rada B. Leto T. L. (2008). Oxidative innate immune defenses by Nox/Duox family NADPH oxidases. *Contrib. Microbiol.* 15 164–187. 10.1159/000136357 18511861 PMC2776633

[B53] Ramos-Kichik V. Mondragón-Flores R. Mondragón-Castelán M. Gonzalez-Pozos S. Muñiz-Hernandez S. Rojas-Espinosa O. (2009). Neutrophil extracellular traps are induced by *Mycobacterium tuberculosis.* *Tuberculosis* 89 29–37. 10.1016/j.tube.2008.09.009 19056316

[B54] Reid S. D. Hong W. Dew K. E. Winn D. R. Pang B. Watt J. (2009). *Streptococcus pneumoniae* forms surface-attached communities in the middle ear of experimentally infected chinchillas. *J. Infect. Dis.* 199 786–794. 10.1086/597042 19434911

[B55] Röhm M. Grimm M. J. D’Auria A. C. Almyroudis N. G. Segal B. H. Urban C. F. (2014). NADPH oxidase promotes neutrophil extracellular trap formation in pulmonary aspergillosis. *Infect. Immun.* 82 1766–1777. 10.1128/IAI.00096-14 24549323 PMC3993456

[B56] Saffarzadeh M. Juenemann C. Queisser M. A. Lochnit G. Barreto G. Galuska S. P. (2012). Neutrophil extracellular traps directly induce epithelial and endothelial cell death: a predominant role of histones. *PLoS ONE* 7:e32366 10.1371/journal.pone.0032366 PMC3289648 22389696

[B57] Saffarzadeh M. Preissner K. T. (2013). Fighting against the dark side of neutrophil extracellular traps in disease: manoeuvres for host protection. *Curr. Opin. Hematol.* 20 3–9. 10.1097/MOH.0b013e32835a0025 23041718

[B58] Saitoh T. Komano J. Saitoh Y. Misawa T. Takahama M. Kozaki T. (2012). Neutrophil extracellular traps mediate a host defense response to human immunodeficiency virus-1. *Cell Host Microbe* 12 109–116. 10.1016/j.chom.2012.05.015 22817992

[B59] Scharrig E. Carestia A. Ferrer M. F. Cédola M. Pretre G. Drut R. (2015). Neutrophil Extracellular Traps are involved in the innate immune response to infection with *Leptospira*. *PLoS Negl. Trop Dis.* 9:e0003927 10.1371/journal.pntd.0003927 PMC4498591 26161745

[B60] Schorn C. Janko C. Latzko M. Chaurio R. Schett G. Herrmann M. (2012). Monosodium urate crystals induce extracellular DNA traps in neutrophils, eosinophils, and basophils but not in mononuclear cells. *Front. Immunol.* 3:27 10.3389/fimmu.2012.0027 PMC3432456 22969769

[B61] Semeraro F. Ammollo C. T. Morrissey J. H. Dale G. L. Friese P. Esmon N. L. (2011). Extracellular histones promote thrombin generation through platelet-dependent mechanisms: involvement of platelet TLR2 and TLR4. *Blood* 118 1952–1961. 10.1182/blood-2011-03-343061 21673343 PMC3158722

[B62] Seper A. Hosseinzadeh A. Gorkiewicz G. Lichtenegger S. Roier S. Leitner D. R. (2013). *Vibrio cholerae* evades neutrophil extracellular traps by the activity of two extracellular nucleases. *PLoS Pathog.* 9:e1003614 10.1371/journal.ppat.1003614 PMC3764145 24039581

[B63] Shanthalingam S. Srikumaran S. (2009). Intact signal peptide of CD18, the beta-subunit of beta2-integrins, renders ruminants susceptible to *Mannheimia haemolytica* leukotoxin. *Proc. Natl. Acad. Sci. U.S.A.* 106 15448–15453. 10.1073/pnas.0906775106 19706410 PMC2741271

[B64] Short K. R. von Köckritz-Blickwede M. Langereis J. D. Chew K. Y. Job E. R. Armitage C. W. (2014). Antibodies mediate formation of neutrophil extracellular traps in the middle ear and facilitate secondary pneumococcal otitis media. *Infect. Immun.* 82 364–370. 10.1128/IAI.01104-13 24191297 PMC3911859

[B65] Spaan A. N. Henry T. van Rooijen W. J. Perret M. Badiou C. Aerts P. C. (2013). The staphylococcal toxin Panton-Valentine Leukocidin targets human C5a receptors. *Cell Host Microbe* 13 584–594. 10.1016/j.chom.2013.04.006 23684309

[B66] Uchiyama S. Döhrmann S. Timmer A. M. Dixit N. Ghochani M. Bhandari T. (2015). Streptolysin o rapidly impairs neutrophil oxidative burst and antibacterial responses to group a *Streptococcus*. *Front. Immunol.* 6:581 10.3389/fimmu.2015.00581 PMC4644796 26635795

[B67] Urban C. F. Ermert D. Schmid M. Abu-Abed U. Goosmann C. Nacken W. (2009). Neutrophil extracellular traps contain calprotectin, a cytosolic protein complex involved in host defense against *Candida albicans*. *PLoS Pathog.* 5:e1000639 10.1371/journal.ppat.1000639 PMC2763347 19876394

[B68] Villanueva E. Yalavarthi S. Berthier C. C. Hodgin J. B. Khandpur R. Lin A. M. (2011). Netting neutrophils induce endothelial damage, infiltrate tissues, and expose immunostimulatory molecules in systemic lupus erythematosus. *J. Immunol.* 187 538–552. 10.4049/jimmunol.1100450 21613614 PMC3119769

[B69] von Köckritz-Blickwede M. Nizet V. (2009). Innate immunity turned inside-out: antimicrobial defense by phagocyte extracellular traps. *J. Mol. Med.* 87 775–783. 10.1007/s00109-009-0481-0 19444424 PMC2707954

[B70] von Köckritz-Blickwede M. von Goldmann O. Thulin P. Heinemann K. Norrby-Teglund A. Rohde M. (2008). Phagocytosis-independent antimicrobial activity of mast cells by means of extracellular trap formation. *Blood* 111 3070–3080. 10.1182/blood-2007-07-104018 18182576

[B71] Wardini A. B. Guimarães-Costa A. B. Nascimento M. T. Nadaes N. R. Danelli M. G. Mazur C. (2010). Characterization of neutrophil extracellular traps in cats naturally infected with feline leukemia virus. *J. Gen. Virol.* 91 259–264. 10.1099/vir.0.014613-0 19793908

[B72] Wartha F. Beiter K. Albiger B. Fernebro J. Zychlinsky A. Normark S. (2007). Capsule and D-alanylated lipoteichoic acids protect *Streptococcus pneumoniae* against neutrophil extracellular traps. *Cell Microbiol.* 9 1162–1171. 10.1111/j.1462-5822.2006.00857.x 17217430

[B73] Wong K. W. Jacobs W. R. Jr. (2013). *Mycobacterium tuberculosis* exploits human interferon γ to stimulate macrophage extracellular trap formation and necrosis. *J. Infect. Dis.* 208 109–119. 10.1093/infdis/jit097 23475311 PMC3666134

[B74] Yipp B. G. Petri B. Salina D. Jenne C. N. Scott B. N. Zbytnuik L. D. (2012). Infection-induced NETosis is a dynamic process involving neutrophil multitasking in vivo. *Nat. Med.* 18 1386–1393. 10.1038/nm.2847 22922410 PMC4529131

[B75] Young R. L. Malcolm K. C. Kret J. E. Caceres S. M. Poch K. R. Nichols D. P. (2011). Neutrophil extracellular trap (NET)-mediated killing of *Pseudomonas aeruginosa*: evidence of acquired resistance within the CF airway, independent of CFTR. *PLoS ONE* 6:e23637 10.1371/journal.pone.0023637 PMC3164657 21909403

[B76] Yousefi S. Gold J. A. Andina N. Lee J. J. Kelly A. M. Kozlowski E. (2008). Catapult-like release of mitochondrial DNA by eosinophils contributes to antibacterial defense. *Nat. Med.* 14 949–953. 10.1038/nm.1855 18690244

[B77] Yousefi S. Mihalache C. Kozlowski E. Schmid I. Simon H. U. (2009). Viable neutrophils release mitochondrial DNA to form neutrophil extracellular traps. *Cell Death Differ.* 16 1438–1444. 10.1038/cdd.2009.96 19609275

[B78] Zinkernagel A. S. Timmer A. M. Pence M. A. Locke J. B. Buchanan J. T. Turner C. E. (2008). The IL-8 protease SpyCEP/ScpC of group A *Streptococcus* promotes resistance to neutrophil killing. *Cell Host Microbe* 4 170–178. 10.1016/j.chom.2008.07.002 18692776 PMC2631432

